# Mass Spectrometry Based Molecular 3D-Cartography of Plant Metabolites

**DOI:** 10.3389/fpls.2017.00429

**Published:** 2017-03-29

**Authors:** Dimitrios J. Floros, Daniel Petras, Clifford A. Kapono, Alexey V. Melnik, Tie-Jun Ling, Rob Knight, Pieter C. Dorrestein

**Affiliations:** ^1^Skaggs School of Pharmacy and Pharmaceutical Sciences, University of California, San Diego, San DiegoCA, USA; ^2^Collaborative Mass Spectrometry Innovation Center, Skaggs School of Pharmacy and Pharmaceutical Sciences, University of California, San Diego, San DiegoCA, USA; ^3^State Key Laboratory of Tea Plant Biology and Utilization, Anhui Agricultural UniversityHefei, China; ^4^Department of Computer Science and Engineering, University of California, San Diego, San DiegoCA, USA; ^5^Center for Microbiome Innovation, University of California, San Diego, San DiegoCA, USA; ^6^Department of Pediatrics, University of California, San Diego, San DiegoCA, USA

**Keywords:** plant metabolomics, imaging mass spectrometry, 3D-imaging, tomato, pepper

## Abstract

Plants play an essential part in global carbon fixing through photosynthesis and are the primary food and energy source for humans. Understanding them thoroughly is therefore of highest interest for humanity. Advances in DNA and RNA sequencing and in protein and metabolite analysis allow the systematic description of plant composition at the molecular level. With imaging mass spectrometry, we can now add a spatial level, typically in the micrometer-to-centimeter range, to their compositions, essential for a detailed molecular understanding. Here we present an LC-MS based approach for 3D plant imaging, which is scalable and allows the analysis of entire plants. We applied this approach in a case study to pepper and tomato plants. Together with MS/MS spectra library matching and spectral networking, this non-targeted workflow provides the highest sensitivity and selectivity for the molecular annotations and imaging of plants, laying the foundation for studies of plant metabolism and plant-environment interactions.

## Introduction

Phototrophs form the foundational layer of energy and nutrient capture in essentially all terrestrial and marine ecosystems (Field et al., 1998). Human society has long relied on both wild and cultivated plants to provide not only dietary energy, essential micronutrients and medicinally useful natural products, but also structural materials and biofuels (Lewis and Elvin-Lewis, 1995; Bouis, 1996; Durrett et al., 2008). Although many studies have investigated the genetic (Koch, 2016), transcriptomic (Imadi et al., 2015), and proteomic properties (Jorrín-Novo et al., 2015) associated with plant tissues, plant metabolites have long been studied only in the context of bioactive metabolic products. The emerging field of metabolomics is opening the door to a global understanding of plant phenotypes at the molecular level (Sumner et al., 2015). With a quarter of plant genes dedicated to metabolism and over 200 thousand unique natural products identified, the detection and mapping of three dimensional distributions of plant chemistries remains underexplored (Dixon and Strack, 2003). For example, what are the chemical differences between tissue types and growth stages? How do these chemotypes behave in comparison to other species? Are the main factors driving the distribution of chemicals over the plant organ structure, vascularization, exposure to sunlight or other environmental gradients as microbial symbionts or pathogens? Understanding these questions at a basic chemical level is an important step to understanding how to engineer plants to better resist erosion and disease, and to improving their efficiency as providers of animal nutrition, feedstocks to industrial processes or the many other roles that plants perform in natural and engineered landscape ecosystems (Yandeau-Nelson et al., 2016).

A wide range of analytical techniques such as NMR and UV or IR spectroscopy, are available for making metabolomic measurements, but suffer from limitations of sensitivity and specificity to individual molecular features (Schripsema, 2010). Unlike spectroscopic techniques, mass spectrometry (MS) allows highly selective and quantitative measurements to be taken of a broad range of chemical families over a large dynamic range. With recent advantages in imaging mass spectrometry (IMS), we can obtain metabolic inventories with spatial information from nm to km scale (Petras et al., 2017). Matrix assisted laser desorption ionization (MALDI) and laser assisted electrospray ionization (LAESI) use laser shots that raster the surface of a sample to generate molecular 2D images, while desorption based DESI and nano-DESI raster continuously flowing solvent droplets across the sample (Watrous et al., 2012; Etalo et al., 2015; Sturtevant et al., 2016). These techniques vary in their requirements for sample preparation, spatial resolution, and compatibility with atmospheric conditions, but are all limited to 2D surface imaging. To create 3D chemical maps, multiple sectioning and reconstruction of 2D layers is required (as in X-ray tomography or magnet resonance imaging). Apart from these techniques, liquid chromatography based mass spectrometry (LC-MS or LC-MS/MS) methods separate complex components of a tissue extracts based on physiochemical properties before their introduction to the mass spectrometer, mostly via electrospray ionization (ESI). This pre-separation enables greater sensitivity and depth of coverage, making it a powerful tool for metabolomic analyses (De Vos et al., 2007). Another advantage of LC-MS based imaging is that the spatial scale is not limited to instrument based dimensions, typically in the cm range. This enables the molecular 3D imaging of humans (Bouslimani et al., 2015) and their habitats (Petras et al., 2016) up to planetary (km) scale. Since the samples are excised, homogenized, and extracted with organic solvents for LC-MS analysis, it is not compatible with direct imaging of plant tissue. However, by generating optical 3D images prior to dissection and recording spatial sampling locations, new visualization tools can map metabolomic features onto the model.

Here we describe an LC-MS based technique for the 3D-mapping of metabolic content of a tomato (*Solanum lycopersicum*) and pepper plant (*Capsicum annuum*), two members of the nightshade family. The protocol and analysis pipeline provide a means for spatially mapping the relative intensities of all metabolites detected through an untargeted LC-MS/MS metabolomics analysis survey of whole plants. Together with global statistical treatments of the data, these ion maps will help investigators to quickly identify patterns and metabolites of interest in their plant systems.

## Experimental Concept

**FIGURE 1:**
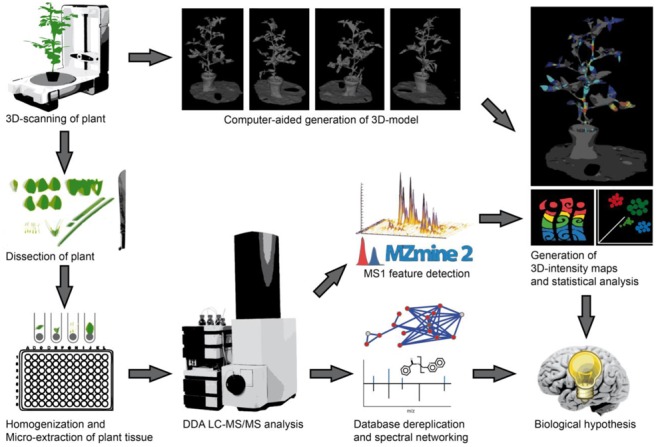
**Workflow from plant to chemical 3D ion intensity maps.** First, the plant is 3D scanned, either using dedicated hardware (for small and/or complex plants) or structure from motion (for larger and/or less complex plants). The 3D model is then generated and used as a template for analysis. Afterwards, the plant is dissected, and metabolites extracted in 96-well plates. Each sample is analyzed by non-targeted LC-MS/MS with data dependent acquisition (DDA). The MS/MS dataset is then dereplicated through MS/MS spectra database comparison and molecular networking is used to relate MS/MS features to one another and to perform chemical identification through spectral proximity. The MS data is used to identify molecular features, which are further linked to MS/MS based identifications in the molecular network. Finally, the location of each sample is mapped back to the 3D structure of the plant to localize the relative intensity of each molecular feature within this 3D structure. Statistical analyses of MS features such as principal coordinates analysis (PCoA) are performed complementing the explicitly spatial map with a dimensionality-reduced abstract map showing intrinsic similarities and differences in the samples.

Our experimental workflow is shown in **Figure 1**. The initial step of our imaging approach is the creation of optical three dimensional models for the representation of the organism to be sampled. For experiments with little plant to plant variation in size and shape it may be suitable to use a single template to map all samples, in this study we generated models of the specific organisms to be used prior to their dissection. Members of the nightshade family, tomato (*S. lycopersicum*) and pepper (*C. annuum*), were therefore purchased from a local nursery. Three dimensional models were generated using a structure from motion software and desktop 3D Scanner to scan the smaller pepper plant. Structure from motion software utilized multiple optical images or photographs from many angles to reconstruct the three dimensional surface of the tomato plant. The tomato plant was placed on a flat surface with minimal background along with several colorful printed reference points to provide consistent points of overlap between photos. The scanner uses infrared lasers as distance finders to accurately capture the surface depth as it is manipulated by a rotating platform, and multiple images are captured and merged by the software. After 3D models had been generated, the aerial plant tissues were dissected, weighed, and quenched in -20°C extraction solvent. Downstream sample preparation involved homogenization and extraction of plant tissues with extraction solvent prior to subsequent evaporation and resuspension for LC-MS/MS analysis. For the LC-MS/MS analysis a 10 min chromatographic separation (UHPLC) method was used, enabling an analysis of 144 samples, e.g., voxel per day. After injecting the samples, molecules were eluted from the reverse phase C_18_ column with a linear gradient of water and acetonitrile and with 0.1% formic acid and infused into hybrid quadrupole-time-of-flight (Q-TOF) mass spectrometer. Once acquired, raw vendor data files were recalibrated with an internal standard and converted to mzXML format. Our first step of LC-MS/MS data analysis was to perform a comparison of the presence and intensity of chromatographic ion peaks in extracted ion maps (EIM) (XIC, e.g., retention time – *m/z* pairs). This process, typically called “feature finding,” can be time consuming and require substantial computational resources, depending on the data size and algorithms used for feature detection and alignment. *MZmine2* (Pluskal et al., 2010), an established tool with easy to use graphical interface was used. After manual inspection of aligned XICs, features were exported as .csv matrix of samples and features with the area under the curve. The feature matrices were used for visualization in principal coordinate analysis (PCoA) with *EMPeror* (Vazquez-Baeza et al., 2013) and for the generation of 3D EIM using *‘ili*. Molecular annotation of features was performed in parallel with MS/MS data by spectra library matching and spectral networking using *GNPS* (Wang et al., 2016). In *GNPS*, the *MScluster* algorithm (Frank et al., 2008) is first used to consolidate identical spectra. All consensus spectra are then compared and scored against each other, and organized into a molecular network depending on their spectral similarity. The network was then visualized in *Cytoscape* (Shannon et al., 2003). Experimental spectra were compared to different reference libraries, including the *ReSpect* library of phytochemical MS/MS spectra (Sawada et al., 2012). As metabolites with similar fragmentation spectra are organized in network clusters, spectral annotations can be propagated throughout the network to provide putative annotations for previously undescribed molecules. Finally, by assigning colors to the network nodes based on their tissue or organisms of origin, one can quickly generate hypotheses about which metabolite families contain chemically biologically distinctive members, and whether these families are non-randomly distributed among plant locations, tissues or species.

## Results and Discussion

**FIGURE 2:**
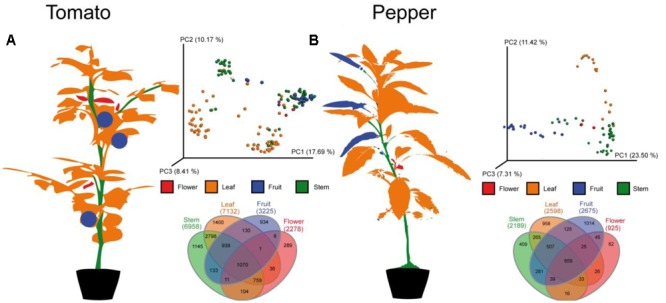
**Molecular distribution across the (A)** tomato and **(B)** pepper plants. In each case, a cartoon of the plant showing the individual patches labeled with the same colors used in the principal coordinates plots and Venn diagrams is shown. The principal coordinates plots show the first three principal axes, using the binary Jaccard’s distance metric. Samples separate by type and the Venn diagrams demonstrate that only a minority of MS features are shared among all parts of the plant (5.5% for tomato, 7.9% for pepper), that stems and leaves share more metabolites than any other pair of samples in both species, and that stems, leaves and fruit each have more unique metabolites than flowers although the rank order of unique metabolites varies between the two plants samples. See **Figure 4** for the distribution of individual metabolites of interest on the 3D structure of the plant.

For an initial global analysis of sample to sample distances, we visualized MS features in the first three dimensions of PCoA space (**Figure 2**) in EMPeror using the binary Jaccard dissimilarity metric (Vazquez-Baeza et al., 2013). Within this space, tissue type distinctions from the global metabolomic level are readily visible. As expected, leaf, flower, fruit, and stem carry distinct metabolic signatures. Some quantitative distinctions may be driven by differences in extraction efficiency between tissue types, like tough stems and high-water content of tomato flesh. However, the qualitative presence/absence of LC-MS features also varies across tissues. In fact less than 10% of LC-MS features are observed in all tissue types, suggesting that untargeted analysis of crude extracts are capturing tissue level chemical diversity. In order to determine to what degree the individual metabolites vary between tissue types it is necessary to begin grouping individual metabolites, identifying those that can be dereplicated and observing families of unknown metabolites.

**FIGURE 3:**
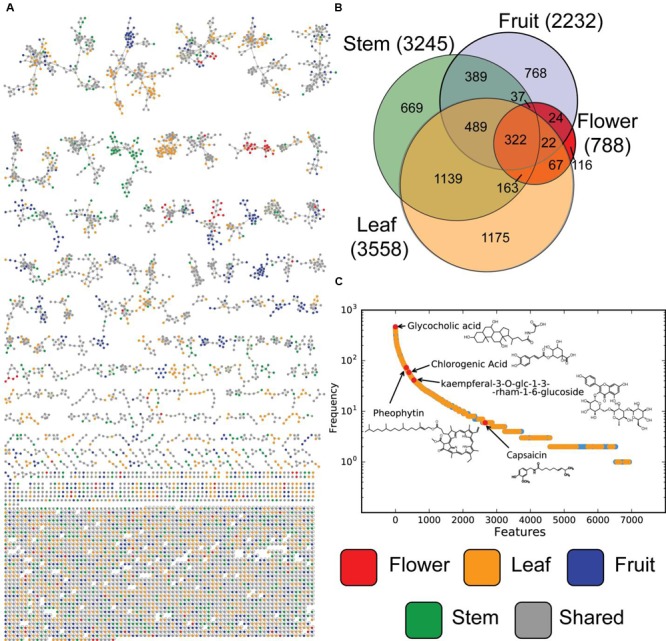
**Molecular MS/MS network and metabolite level overlap. (A)** Product ion spectra of all tissues samples, were analyzed based on their spectral proximity with GNPS. Every node represents a single or set of identical spectra, linked to similar MS/MS spectra. The different tissue origins of the spectra are indicated by different node colors. Nodes originating from solvent and/or system blanks were subtracted. In **(B)** Venn diagrams of the occurrence of unique and shared MS/MS spectra between different tissue types are shown. In **(C)** the frequency plot of clusters of MS/MS spectra (represented as nodes in the network) vs. samples in which this MS/MS spectrum is observed.

LC-MS features are the first level of mass spectrometric data, but molecular fragmentation (MS/MS) spectra were also collected. These fragmentation patterns contain chemical information about individual metabolites that can be compared using spectra library comparison and spectral networking (Wang et al., 2016). This allows metabolites to be grouped into families of similar compounds as well as being matched against libraries of known compounds (Nguyen et al., 2013). From the resulting molecular network the background nodes, shared with solvent and system blanks, were subtracted, leaving in total 5598 nodes (**Figure 3A**). Of those, 92 consensus spectra matched thereby to spectra from the libraries within a mass error of 20 ppm, yielding a 1.6% annotation rate. When categorized by tissue type (**Figure 3B**), MS/MS nodes show similar distributions as MS features (**Figure 2**), with stem and leaf metabolites making up a large number of total amount of nodes. Fruit and flower tissues contribute many unique signals, while a relatively small number of metabolites are universally detected in all tissues. Matches to library data are present all along this distribution (**Figure 3C**), providing evidence that the untargeted metabolomics equally sampled a wide range of compounds and that these were represented within the library. Molecular networking performs especially well in identifying uniquely expressed metabolites in a survey (Floros et al., 2016; Nguyen et al., 2016). It is clear that in our molecular network (**Figure 3A**), with background spectra removed, are multiple molecular families (connected network components) which are either unique to or dominated by samples from specific tissue types.

In **Figure 3C** the distribution of the frequency at which metabolites are observed at the MS/MS level ranges from the observations for the internal standard, glycocholic acid, in every sample to pheophytin, chlorophyll without the central Mg^2+^ ion (Lambers et al., 2008), which was observed in more than 60 samples from stem and leafs, which is not surprising as it forms an essential part of photosynthesis. Chlorogenic acid, on the other hand, an intermediate of the biosynthesis of lignin (Boerjan et al., 2003), an important organic polymer and structural building block of plants, was observed in several redundant nodes, originating from more than 41 samples from stem and leaf. Spectral redundancy can be typically overserved in molecular networks, especially for frequently observed compounds. The reasons therefore can be the differences in abundance of precursors which results in presents or absence of low intensity MS/MS fragments and thus classification in different consensus spectra. The flavonoid, kaempferol-3-*O*-glucoside, a typical phytochemical which has been studied in the context of antioxidant and anti-inflammatory activity (Parveen et al., 2007) was present in total 34 samples, but mostly in leaves. On the low frequent side, nodes matching to spectra from capsaicin were only observed in eight samples originating from fruits from the pepper plant.

**FIGURE 4:**
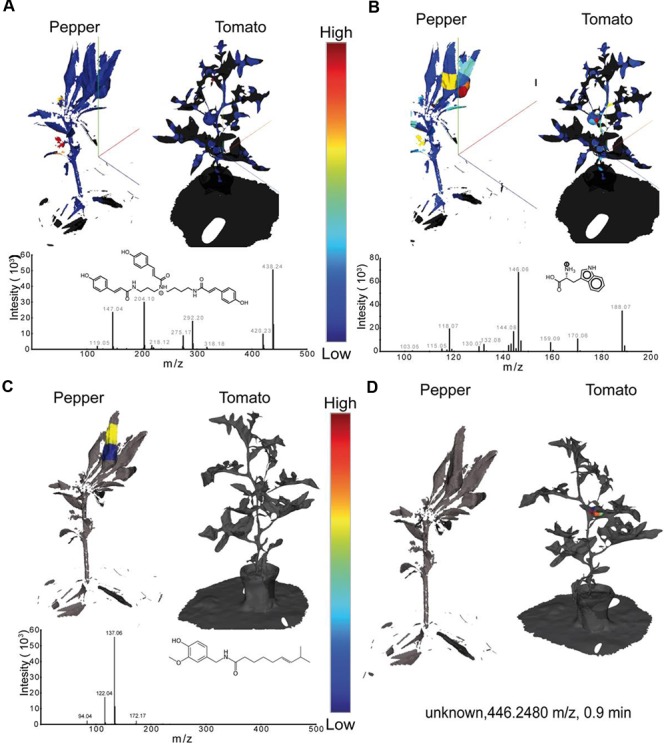
**Extracted 3D ion maps of a pepper and tomato plant.** The spatial distribution of **(A)** tri-coumaroyl- spermidine, **(B)** tryptophan, **(C)** Capsaicin, and **(D)** an unknown compound are shown. Relative concentrations of each *m/z* - retention-time feature are displayed with a linear color gradient (blue – red) in the extracted ion maps of both plants. All MS features detected in our study can be found in the supporting information and can be visualized by drag and drop of the .stl and .csv files in ‘*ili* (ili-toolbox.github.io).

Once data at the global levels has indicated that tissue level distinctions and tissue specific metabolite families are present, the visualization of individual metabolites was achieved with the generation of EIMs (**Figure 4**). Visualization in ‘*ili* allows investigators to cycle through the distributions of each individual metabolite. This takes advantage of the brain’s enormous capacity to absorb visual information and detect patterns; EIMs allow rapid hypothesis generation and potential biomarker selection. Comparing single metabolite patterns to known physiological and pathological patterns can provide insight into the biological function of certain metabolites in many systems. **Figure 4** shows exemplary EIMs of the above-mentioned metabolite features. Once present in *‘ili*, the creation of an EIM can be performed instantly with every molecular feature present in the .csv file. In **Figure 4A**, the distribution of tri-coumaroyl spermidine can be seen. The strongest abundance can be overserved at both, the flowers of the pepper and the tomato plant. These findings are in line with the literature, where coumaroylated spermidines have been reported to be common in floral organs (Werner et al., 1995), in particular in tomatoes (Larbat et al., 2014). Phenolic amines, such as tri-coumaroylated spermidine, have thereby been studied mainly in the context of plant defense and biotic aggression (Elejalde-Palmett et al., 2015) which might be an important molecular factor for future crop breeding. In **Figure 4B** the spatial distribution of tryptophan, a canonical amino acid, is shown. As tryptophan is common in basically all living organism it’s detection is not too surprising. However, tryptophan is a biosynthetic precursor of indole acetic acid – an auxin important for fruit development (Ljung et al., 2002). Hence, it’s interesting to observe that the relative concentration of tryptophan shows the highest abundance in the tomato and pepper fruits, which can be important to understand differences in fruit maturing processes, particularly of agricultural important species. **Figure 4C** shows the spatial distribution of capsaicin which was found only in the fruit of the pepper plant. In **Figure 4D** the spatial distribution of an unknown metabolite with the mass 446.2480 m/z is shown which is only observed in the tomato. Although it is unknown now, it might be identified and automatically annotated through the “living data” in GNPS or could be subjected to further targeted structural and functional investigations.

Summarizing, LC-MS based 3D imaging of plants introduces a new tool to spatially visualize small molecules in plants and thus to understand phenotypic and genetic differences on a metabolite level. Paired with spatial high resolution imaging methods such as MALDI and other modalities, we anticipate this technique will provide the base for most comprehensive molecular models of complete plants and their interactions with their environment and microorganisms.

## Experimental Procedure

### Plants

Two individual plants, tomato (*S. lycopersicum*) and pepper (*C. annuum*), were purchased from a local nursery (Green Gardens Nursery, San Diego, CA, USA) in October 2015.

### Model Generation

3D models were generated using the structure-from-motion software 123Dcatch (Autodesk, San Rafael, CA, USA) for the larger tomato plant, and the 3D Scanner *Ultra HD* (NextEngine, Santa Monica, CA, USA) for portions of the pepper plant. Both platforms provide output file in stl formats, which are then used with the ‘*ili* toolbox.

### Sectioning, Processing, and Extraction

All aerial plant tissues parts were dissected manually into sections with a size convenient for extraction in a 1.5 mL reaction tube. Each tissue section was weighed, with masses ranging from 0.01 to 0.03 grams. Tissues were placed in round-bottom solvent resistant tubes along with a 5 mm stainless steel bead and 500 microliters of extraction solvent (2:2:1 ethyl acetate:methanol:water) and were disrupted by shaking at 30 Hz for 15 min in a *TissueLyser* (Qiagen, Hilden, Germany). Plant tissues were allowed to extract for at least 8 h at -20°C. Tubes were then centrifuged briefly to pellet solid material and the supernatants were transferred to 1 mL deep 96-well plates and stored at -20°C until being evaporated to dryness in a *Speedvac* vacuum centrifuge (Thermo, Bremen, Germany). Samples were then resuspended in injection solvent (9:1 methanol:water containing 10 mM glycocholic acid as internal standard) by sonication of the entire plate for 5 min. Again, insoluble particulates were pelleted by centrifugation at 20,000 *g* for 10 min. Finally, 100 microliters of extracted solvent was transferred to polypropylene 0.3 mL 96-well plates (NUNC, Roskilde, Denmark). Plates were stored at -80°C until LC-MS analysis.

### LC-MS Analysis

All sample extracts were subjected to chromatographic separation with an Agilent *1290 Infinity* UHPLC system (Agilent, Waldbronn, Germany). Separations were achieved using a flow rate of 0.5 mL/min with an 1.7 micron C18 (50 × 2.1 mm) *Kinetex* UHPLC column (Phenomenex, Torrance, CA, USA) held at 30°C. The gradient was 0–0.5 min 5% B, 0.5–8 min 5–100% B, 8–11 min 100% B, 11–11.5 min 100–5% B, 11.5–12 min 5% B, where solvent A is water with 0.1% formic acid (v/v) and solvent B is acetonitrile with 0.1% formic acid (v/v). MS/MS experiments were performed on a *Maxis* QTOF mass spectrometer (Bruker, Bremen, Germany) equipped with an ESI source in positive mode with mass range 100–2000 m/z. Before analysis, the instrument was externally calibrated to 1.0 ppm mass accuracy with ESI-L Low Concentration Tuning Mix (Agilent, Waldbronn, Germany). During the analysis m/z 622.029509, was used as an internal calibrant. Instrument parameters were as follows: nebulizer gas (N_2_) pressure, two bar; Capillary voltage, 4500 V; ion source temperature 200°C; dry gas 9 L/min; spectra rate, 2 Hz. MS/MS fragmentation of the five most abundant ions per spectrum was performed with adaptive collision energy and acquisition time based on precursor ion properties (see Supplementary Materials). With total cycle time of 3 s, ions were excluded from reselection after three spectra and released after 20 s. An exclusion list was used to prevent sampling of the lock mass. All LC-MS analyses were controlled by *Hystar* and *Otof Control* software packages (Bruker, Bremen, Germany). Raw data was then converted to .mzXML format.

### Data Processing and Multivariable Statistical Analysis

For the global statistical analysis, the MS/MS scans were removed from the original mzXML files using *MSConvert* (Deutsch et al., 2015) to decrease the total file and speed up down-stream processing. Feature extraction was performed with *MZmine2* (Pluskal et al., 2010) with a signal threshold of 2.0e^6^ and 0.3 s minimum peak width. The mass tolerance was set to 10 ppm and the maximum allowed retention time deviation was set to 10 s. For chromatographic deconvolution the baseline cutoff algorithm with 1.0e4 signal threshold was used. Maximum peak width was set to 2 min. After isotope peak removal, the peak lists of all samples were aligned with the above-mentioned retention time and mass tolerances. After the creation of a feature matrix containing the feature retention times, exact mass and peak areas of the corresponding extracted ion chromatograms, metadata of the samples (plant type, tissue type, and spatial coordinates) was added. For the PCoA the signal intensity of the features was normalized to the total ion current of all samples. The PCoA plots were generated with the binary Jaccard dissimilarity metric using the in-house tool *ClusterApp* and visualized in *EMPeror* (Vazquez-Baeza et al., 2013). Venn diagrams of shared MS1 features were generated using *InteractiVenn* (Heberle et al., 2015).

### Molecular Networking

MS/MS data was analyzed with *GNPS* (Wang et al., 2016). Therefore, the data was filtered by removing all MS/MS peaks within a 17 Da window of the precursor m/z and MS/MS spectra were filtered by choosing only the top 6 peaks in 50 Da windows. The data was then clustered with MS-Cluster (Frank et al., 2008), with a precursor mass tolerance of 0.02 Da and a MS/MS fragment ion tolerance of 0.02 Da. Consensus spectra with less than 2 spectra were discarded. A spectral network was then created with a minimum spectral similarity of cosine 0.7 and more than 4 matched peaks. Only top 10 edged connecting one note were kept in the network. Consensus spectra were searched against the *GNPS* spectral library as well as *Massbank, ReSpect, HMDB*, and *NIST14* (Forsythe and Wishart, 2009; Horai et al., 2010; Sawada et al., 2012; Stein, 2014; Wang et al., 2016) with a precursor mass tolerance of 0.02 Da and a MS/MS fragment ion tolerance of 0.02 Da. Nodes originating from solvent and/or system blanks were subtracted. Venn/Euler diagrams of MS/MS features from *GNPS* were generated using the Venn and Euler Diagrams app (Heuer et al., 2016) in the *Cytoscape 3* environment (Shannon et al., 2003).

### 3D Mapping and Visual Analysis

We used the *GeoMagic 3D* visualization and editing software (3D Systems, Rock Hill, SC, USA) to clean up the automatically generated 3D mesh objects from the .stl-format files. This software was also used to identify coordinates corresponding to each tissue sample. Each pair of filenames and coordinates was associated with their corresponding MS features and MS/MS IDs in a .csv-format table. These tables were uploaded, along with the .stl 3D model, to the visualization tool ‘*ili* (Alexandrov, 2016).

## Data Accessibility

All LC-MS/MS data can be found on the Mass spectrometry Interactive Virtual Environment (MassIVE) at https://massive.ucsd.edu/ with the identifiers MSV000079448, MSV000079447, and MSV000079446. 3D .stl files and corresponding ion intensities in .csv format are available in the supporting information of this article.

## Author Contributions

DF, DP, and PD designed the study. DF, AM, CK, and T-JL collected the samples. DF and AM acquired the MS data. DF, DP, CK, and AM performed the 3D mapping. DF and DP processed and analyzed the data. DF, DP, RK, and PD wrote the manuscript. All authors discussed and approved the manuscript.

## Conflict of Interest Statement

The authors declare that the research was conducted in the absence of any commercial or financial relationships that could be construed as a potential conflict of interest.

## References

[B1] Alexandrov T. (2016). *Ili-Toolbox/ili.* Available at: https://github.com/ili-toolbox/ili [accessed April 27, 2016].

[B2] Boerjan W. Ralph J. Baucher M. (2003). Lignin biosynthesis. *Annu. Rev. Plant Biol.* 54 519–546. 10.1146/annurev.arplant.54.031902.134938 14503002

[B3] Bouis H. (1996). Enrichment of food staples through plant breeding: a new strategy for fighting micronutrient malnutrition. *Nutr. Rev.* 54 131–137. 10.1111/j.1753-4887.1996.tb03915.x 8783879

[B4] Bouslimani A. Porto C. Rath C. M. Wang M. Guo Y. Gonzalez A. (2015). Molecular cartography of the human skin surface in 3D. *Proc. Natl. Acad. Sci. U.S.A.* 112 E2120–E2129. 10.1073/pnas.1424409112 25825778 PMC4418856

[B5] De Vos R. C. Moco S. Lommen A. Keurentjes J. J. Bino R. J. Hall R. D. (2007). Untargeted large-scale plant metabolomics using liquid chromatography coupled to mass spectrometry. *Nat. Protoc.* 2 778–791. 10.1038/nprot.2007.95 17446877

[B6] Deutsch E. W. Mendoza L. Shteynberg D. Slagel J. Sun Z. Moritz R. L. (2015). Trans-Proteomic Pipeline, a standardized data processing pipeline for large-scale reproducible proteomics informatics. *Proteomics Clin. Appl.* 9 745–754. 10.1002/prca.201400164 25631240 PMC4506239

[B7] Dixon R. A. Strack D. (2003). Phytochemistry meets genome analysis, and beyond. *Phytochemistry* 62 815–816. 10.1016/S0031-9422(02)00712-4 12590109

[B8] Durrett T. P. Benning C. Ohlrogge J. (2008). Plant triacylglycerols as feedstocks for the production of biofuels. *Plant J.* 54 593–607. 10.1111/j.1365-313X.2008.03442.x 18476866

[B9] Elejalde-Palmett C. de Bernonville T. D. Glevarec G. Pichon O. Papon N. Courdavault V. (2015). Characterization of a spermidine hydroxycinnamoyltransferase in *Malus domestica* highlights the evolutionary conservation of trihydroxycinnamoyl spermidines in pollen coat of core Eudicotyledons. *J. Exp. Bot.* 66 7271–7285. 10.1093/jxb/erv423 26363642

[B10] Etalo D. W. De Vos R. C. Joosten M. H. Hall R. D. (2015). Spatially resolved plant metabolomics: some potentials and limitations of laser-ablation electrospray ionization mass spectrometry metabolite imaging. *Plant Physiol.* 169 1424–1435. 10.1104/pp.15.01176 26392264 PMC4634093

[B11] Field C. B. Behrenfeld M. J. Randerson J. T. Falkowski P. (1998). Primary production of the biosphere: integrating terrestrial and oceanic components. *Science* 281 237–240. 10.1126/science.281.5374.237 9657713

[B12] Floros D. J. Jensen P. R. Dorrestein P. C. Koyama N. (2016). A metabolomics guided exploration of marine natural product chemical space. *Metabolomics* 12 145 10.1007/s11306-016-1087-5 PMC5556696 28819353

[B13] Forsythe I. J. Wishart D. S. (2009). Exploring human metabolites using the human metabolome database. *Curr. Protoc. Bioinformatics* Chapter 14 Unit14.8 10.1002/0471250953.bi1408s25 19274632

[B14] Frank A. M. Bandeira N. Shen Z. Tanner S. Briggs S. P. Smith R. D. (2008). Clustering millions of tandem mass spectra. *J. Proteome Res.* 7 113–122. 10.1021/pr070361e 18067247 PMC2533155

[B15] Heberle H. Meirelles G. V. da Silva F. R. Telles G. P. Minghim R. (2015). InteractiVenn: a web-based tool for the analysis of sets through Venn diagrams. *BMC Bioinformatics* 16:1 10.1186/s12859-015-0611-3 PMC4455604 25994840

[B16] Heuer M. Smoot M. Venn Euler Diagrams (2016). Available at: http://apps.cytoscape.org/apps/vennandeulerdiagrams [accessed Apr 27, 2016]

[B17] Horai H. Arita M. Kanaya S. Nihei Y. Ikeda T. Suwa K. (2010). MassBank: a public repository for sharing mass spectral data for life sciences. *J. Mass Spectrom.* 45 703–714. 10.1002/jms.1777 20623627

[B18] Imadi S. R. Kazi A. G. Ahanger M. A. Gucel S. Ahmad P. (2015). Plant transcriptomics and responses to environmental stress: an overview. *J. Genet.* 94 525–537. 10.1007/s12041-015-0545-6 26440096

[B19] Jorrín-Novo J. V. Pascual J. Sánchez-Lucas R. Romero-Rodríguez M. C. Rodríguez-Ortega M. J. Lenz C. (2015). Fourteen years of plant proteomics reflected in Proteomics: moving from model species and 2DE-based approaches to orphan species and gel-free platforms. *Proteomics* 15 1089–1112. 10.1002/pmic.201400349 25487722

[B20] Koch L. (2016). Plant genomics: 1001 genomes and epigenomes. *Nat. Rev. Genet.* 17 503–503. 10.1038/nrg.2016.99 27452113

[B21] Lambers H. Chapin I. I. I. F. S. Pons T. L. (eds) (2008). “Photosynthesis,” in *Plant Physiological Ecology* (New York, NY: Springer) 11–99. 10.1007/978-0-387-78341-3_2

[B22] Larbat R. Paris C. Le Bot J. Adamowicz S. (2014). Phenolic characterization and variability in leaves, stems and roots of Micro-Tom and patio tomatoes, in response to nitrogen limitation. *Plant Sci.* 224 62–73. 10.1016/j.plantsci.2014.04.010 24908507

[B23] Lewis W. H. Elvin-Lewis M. P. (1995). Medicinal plants as sources of new therapeutics. *Ann. Mo. Bot. Gard.* 82 16–24. 10.2307/2399976

[B24] Ljung K. Hull A. K. Kowalczyk M. Marchant A. Celenza J. Cohen J. D. (2002). “Biosynthesis, conjugation, catabolism and homeostasis of indole-3-acetic acid in *Arabidopsis thaliana*,” in *Auxin Molecular Biology* eds Rechenmann C. P. Hagen G. (New York, NY: Springer) 249–272. 12036253

[B25] Nguyen D. D. Melnik A. V. Koyama N. Lu X. Schorn M. Fang J. (2016). Indexing the *Pseudomonas* specialized metabolome enabled the discovery of poaeamide B and the bananamides. *Nat. Microbiol.* 2:16197 10.1038/nmicrobiol.2016.197 PMC5538791 27798598

[B26] Nguyen D. D. Wu C. H. Moree W. J. Lamsa A. Medema M. H. Zhao X. (2013). MS/MS networking guided analysis of molecule and gene cluster families. *Proc. Natl. Acad. Sci. U.S.A.* 110 E2611–E2620. 10.1073/pnas.1303471110 23798442 PMC3710860

[B27] Parveen Z. Deng Y. Saeed M. K. Dai R. Ahamad W. Yu Y. H. (2007). Antiinflammatory and analgesic activities of *Thesium chinense* Turcz extracts and its major flavonoids, kaempferol and kaempferol-3-O-glucoside. *Yakugaku Zasshi* 127 1275–1279. 10.1248/yakushi.127.1275 17666881

[B28] Petras D. Jarmusch A. K. Dorrestein P. C. (2017). From single cells to our planet–Recent advances in using mass spectrometry for spatially resolved metabolomics. *Curr. Opin. Chem. Biol.* 36 24–31. 10.1016/j.cbpa.2016.12.018 28086192

[B29] Petras D. Nothias L. F. Quinn R. A. Alexandrov T. Bandeira N. Bouslimani A. (2016). Mass spectrometry-based visualization of molecules associated with human habitats. *Anal. Chem.* 88 10775–10784. 10.1021/acs.analchem.6b03456 27732780 PMC6326777

[B30] Pluskal T. Castillo S. Villar-Briones A. Oresic M. (2010). MZmine 2: modular framework for processing, visualizing, and analyzing mass spectrometry-based molecular profile data. *BMC Bioinformatics* 11:395 10.1186/1471-2105-11-395 PMC2918584 20650010

[B31] Sawada Y. Nakabayashi R. Yamada Y. Suzuki M. Sato M. Sakata A. (2012). RIKEN tandem mass spectral database (ReSpect) for phytochemicals: a plant-specific MS/MS-based data resource and database. *Phytochemistry* 82 38–45. 10.1016/j.phytochem.2012.07.007 22867903

[B32] Schripsema J. (2010). Application of NMR in plant metabolomics: techniques, problems and prospects. *Phytochem. Anal.* 21 14–21. 10.1002/pca.1185 19904731

[B33] Shannon P. Markiel A. Ozier O. Baliga N. S. Wang J. T. Ramage D. (2003). Cytoscape: a software environment for integrated models of biomolecular interaction networks. *Genome Res.* 13 2498–2504. 10.1101/gr.1239303 14597658 PMC403769

[B34] Stein S. (2014). *The NIST 14 Mass Spectral Library.* Gaithersburg, MD: National Institute of Standards and Technology.

[B35] Sturtevant D. Lee Y.-J. Chapman K. D. (2016). Matrix assisted laser desorption/ionization-mass spectrometry imaging (MALDI-MSI) for direct visualization of plant metabolites in situ. *Curr. Opin. Biotechnol.* 37 53–60. 10.1016/j.copbio.2015.10.004 26613199

[B36] Sumner L. W. Lei Z. Nikolau B. J. Saito K. (2015). Modern plant metabolomics: advanced natural product gene discoveries, improved technologies, and future prospects. *Nat. Prod. Rep.* 32 212–229. 10.1039/c4np00072b 25342293

[B37] Vazquez-Baeza Y. Pirrung M. Gonzalez A. Knight R. (2013). EMPeror: a tool for visualizing high-throughput microbial community data. *Gigascience* 2:16 10.1186/2047-217X-2-16 PMC4076506 24280061

[B38] Wang M. Carver J. J. Phelan V. V. Sanchez L. M. Garg N. Peng Y. (2016). Sharing and community curation of mass spectrometry data with global natural products social molecular networking. *Nat. Biotechnol.* 34 828–837. 10.1038/nbt.3597 27504778 PMC5321674

[B39] Watrous J. Roach P. Alexandrov T. Heath B. S. Yang J. Y. Kersten R. D. (2012). Mass spectral molecular networking of living microbial colonies. *Proc. Natl. Acad. Sci. U.S.A.* 109 E1743–E1752. 10.1073/pnas.1203689109 22586093 PMC3387089

[B40] Werner C. Hu W. Lorenzi-Riatsch A. Hesse M. (1995). Di-coumaroylspermidines and tri-coumaroylspermidines in anthers of different species of the genus Aphelandra. *Phytochemistry* 40 461–465. 10.1016/0031-9422(95)00288-I

[B41] Yandeau-Nelson M. Lauter N. Zabotina O. (2016). Advances in metabolomic applications in plant genetics and breeding). *CAB Rev.* 10 1–15.

